# KCF-S: KEGG Chemical Function and Substructure for improved interpretability and prediction in chemical bioinformatics

**DOI:** 10.1186/1752-0509-7-S6-S2

**Published:** 2013-12-13

**Authors:** Masaaki Kotera, Yasuo Tabei, Yoshihiro Yamanishi, Yuki Moriya, Toshiaki Tokimatsu, Minoru Kanehisa, Susumu Goto

**Affiliations:** 1 Bioinformatics Center, Institute for Chemical Research, Kyoto University, Gokasho, Uji, Kyoto 611-0011, Japan; 2 PRESTO, Japan Science and Technology Agency, Kawaguchi, Saitama 332-0012, Japan; 3 Division of System Cohort, Medical Institute of Bioregulation, Kyushu University, 3-1-1 Maidashi, Higashi-ku, Fukuoka, Fukuoka 812-8582, Japan; 4 Institute for Advanced Study, Kyushu University, 6-10-1, Hakozaki, Higashi-ku, Fukuoka, Fukuoka 812-8581, Japan

## Abstract

**Background:**

In order to develop hypothesis on unknown metabolic pathways, biochemists frequently rely on literature that uses a free-text format to describe functional groups or substructures. In computational chemistry or cheminformatics, molecules are typically represented by chemical descriptors, *i.e*., vectors that summarize information on its various properties. However, it is difficult to interpret these chemical descriptors since they are not directly linked to the terminology of functional groups or substructures that the biochemists use.

**Methods:**

In this study, we used KEGG Chemical Function (KCF) format to computationally describe biochemical substructures in seven attributes that resemble biochemists' way of dealing with substructures.

**Results:**

We established KCF-S (KCF-and-Substructures) format as an additional structural information of KCF. Applying KCF-S revealed the specific appearance of substructures from various datasets of molecules that describes the characteristics of the respective datasets. Structure-based clustering of molecules using KCF-S resulted the clusters in which molecular weights and structures were less diverse than those obtained by conventional chemical fingerprints. We further applied KCF-S to find the pairs of molecules that are possibly converted to each other in enzymatic reactions, and KCF-S clearly improved predictive performance than that presented previously.

**Conclusions:**

KCF-S defines biochemical substructures with keeping interpretability, suggesting the potential to apply more studies on chemical bioinformatics. KCF and KCF-S can be automatically converted from Molfile format, enabling to deal with molecules from any data sources.

## Background

By analogy with orphan genes in genomic studies [1], metabolites that are not yet known how they are synthesized or degraded are referred to as "orphan metabolites" [2]. In contrast to the increasing number of the successful genome projects, there still remain many orphan metabolites. For example, it is estimated that plants produce over 200,000 secondary metabolites [3] that are not directly involved in the primary metabolism and whose absence is not normally lethal. Kanaya and colleagues have been collecting 50,897 metabolites, and the chemical structures and metabolite-species relationships are publicly available in KNApSAcK database [4]. Some of them are known to function as toxins defending the organisms against pathogens, parasites and predators [5]. The physiological roles of many such metabolites are still unknown; however, some of them are important sources of drugs and industrial materials.

Many studies have been conducted for the experimental identification of the biosynthetic pathways for such orphan metabolites. In many cases when the chemical structure of the final products are apparent, the structures of intermediates and the chemical transformations (enzyme reactions) are hypothesized by the biochemists' expert knowledge based on organic chemistry and biochemistry, and the hypothesis are verified by the experiments such as liquid chromatography / mass spectrometry (LC/MS) and nuclear magnetic resonance (NMR). In order to develop these hypothesis, biochemists frequently rely on literature that uses a free-text format to describe functional groups or substructures. Thus, a direct link between the names and (sub)structures of compounds and the functional groups contained within them is important.

Some computational studies conduct *de novo *metabolic pathway reconstruction, *i.e*., automated generation of hypothetical metabolic pathway [6-15]. Among them, a group of methods deal with the problem of "enzymatic-reaction likeness", *i.e*., whether or not a compound-compound pair is possibly converted to each other by enzymatic reactions [11-15].

However, the (sub)structures of metabolites in these methods were represented computationally, and it is sometimes difficult to interpret such substructures because they are not designed as similar with the substructures that biochemists usually deal with.

In computational chemistry or cheminformatics, molecules are typically represented by chemical descriptors, *i.e*., vectors that summarize information on its various properties. One group of such descriptors is called chemical fingerprints, which are bit strings that encode the presence or absence of substructures and various physicochemical properties in a molecule into binary variables. Many fingerprints have been designed for the rapid search of molecules, especially for pharmaceutical purposes, from a large amount of molecules in databases. Representative fingerprints include MACCS fingerprint and PubChem fingerprint, and they can be calculated by many freewares such as Chemistry Development Kit [16]. These fingerprints can be used as an input of various machine learning tasks that include similarity search, classification and regression.

These fingerprints only represent presence or absence of substructures, so the numbers of the substructures are not taken into account. This means that, even if a substrate contains two carboxyl groups and one of them turned into an amide group, these fingerprints only detects the generation of the amide group but do not detect the elimination of a carboxyl group. Moreover, they can not distinguish many functional groups (such as aldehyde R-(C=O)-H and carboxylate R-(C=O)-OH), which are obviously different from the viewpoint of organic reactions because of the difference in reactivities. Therefore, discriminating these two types of carbon when comparing molecules is reasonable. Therefore, a more suitable data representation would be needed for improving the prediction accuracy and interpretability for the *de novo *metabolic pathway reconstruction.

In this study, we designed KCF-S (KEGG Chemical Function and Substructures), a new chemical data format describing the numbers of different levels of functional groups and substructures that are related to chemical structure conversion in enzyme reactions. This is an extension of the KCF (KEGG Chemical Function) format that we published in 2003 [17]. KCF takes into account physicochemical environmental properties of atoms by assigning well-detailed vertex labels, named as KEGG Atom Types, which distinguish important functional groups such as carboxylate and aldehyde. In KCF-S, substructures are computationally defined using seven attributes: atom, bond, triplet, vicinity, ring, skeleton, and inorganic. These definitions are designed so that many of them can be explained by the words in organic chemistry or biochemistry.

The proposed KCF-S can be used for many applications. As the first application, we used KCF-S for the structure-based clustering of molecules in a large scale database. As the second application, we used KCF-S for the *de novo *metabolic pathway reconstruction for in the "reaction-filling framework", and showed clearly improved predictive performance compared with the previous method. KCF-S has more potential to apply many other purposes, such as pharmacogenomic analysis and enzyme informatics.

## Data

### KEGG and KNApSAcK as chemical structure databases

We obtained chemical structure of molecules in KEGG [18] and KNApSAcK [4] databases in the Molfile format. Kyoto Encyclopedia of Genes and Genomes (KEGG, http://www.kegg.jp/) is a database resource for understanding high-level functions and utilities of the biological system, which contains a variety of sub-databases such as KEGG COMPOUND and KEGG DRUG. KEGG COMPOUND collects small molecules and other chemical substances (17,012 compounds as of June 2013) that are relevant to biological systems. Each KEGG COMPOUND entry is identified by the ID number consisting of the letter "C" and the five digit numerals (such as C00047 for L-lysine). KEGG DRUG is a comprehensive drug information resource for approved drugs in Japan, USA, and Europe unified based on the chemical structures and/or the chemical components (9,915 drugs as of June 2013), and associated with target, metabolizing enzyme, and other molecular interaction network information. Each KEGG DRUG entry is identified by the ID number consisting of the letter "D" and the five digit numerals (such as D08163 for meclozine, an H1-receptor antagonist). KNApSAcK database (http://kanaya.naist.jp/KNApSAcK/) is a comprehensive species-metabolite relationship database that contains 50,897 metabolites and 109,976 metabolite-species relationships (as of May 2013). Each KNApSAcK entry is identified by ID consisting of the letter "C" and the eight digit numerals (C00036189 for pectinolide A, a secondary metabolite taken from plant *Hyptis pectinata*).

### KEGG Chemical Function (KCF) format

**Figure 1:**
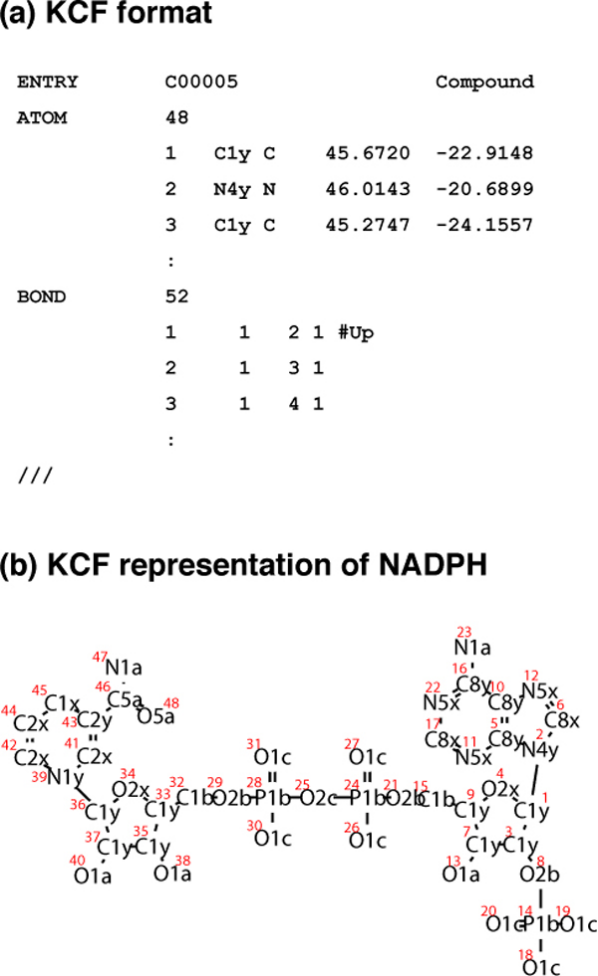
**KEGG Chemical Function (KCF) format**. (a) KEGG Chemical Function (KCF) format of NADPH. KCF format has three sections; ENTRY, ATOM and BOND. ENTRY section describes the KEGG ID and the type of the entry. ATOM section describes the numbering of the atoms, KEGG Atom Types for the labels on the atoms, atomic species (C for carbon, N for nitrogen, etc), and 2D coordinates of the atoms. BOND section describes the numbering of the bonds, the numbering of the two atoms in the bond, and the bond order, and steric configuration of the bond. (b) KCF representation of NADPH. Molecules are represented as graph structures, where nodes represent atoms labeled with KEGG Atom Types.

KEGG Chemical Function (KCF) format, one of the chemical structure file format, has been defined and published in Hattori *et al*., 2003 [17], where molecules (chemical compounds) are represented as graphs consisting of atoms as vertices and bonds as edges (Figure 1). The vertices (atoms) of KCF are labeled by the 68 KEGG Atom types (Table 1), describing the detailed information of atomic properties such as functional groups. The the three-letter labels of the KEGG atoms, such as "C1a" meaning a methyl carbon, represent the hierarchical classification of atom environments. In this study, up to the first, the second, and the third letters of the labels are referred to as the "atom species", the "atom classes", and the "KEGG atoms", respectively. Any organic molecule structure can be converted into KCF, as long as it is described in the Molfile format.

**Table 1:** KEGG Atom Types.

	Carbon atoms
C1a	R-CH3 / methyl
C1b	R-CH2-R / methylene
C1c	R-CH(-R)-R / tertiary carbon
C1d	R-C(-R)2-R / quaternary carbon
C1x	ring-CH2-ring / methylene in ring
C1y	ring-CH(-R)-ring / tertiary carbon in ring
C1z	ring-C(-R)2-ring / quaternary carbon in ring

C2a	R=CH2 / alkenyl terminus carbon
C2b	R=CH-R / alkenyl secondary carbon
C2c	R=C(-R)2 / alkenyl tertiary carbon
C2x	ring-CH=ring / alkenyl secondary carbon in ring
C2y	ring-C(-R)=ring or ring-C(=R)-ring / alkenyl tertiary carbon in ring

C3a	R#CH / alkynyl terminus carbon
C3b	R#C-R / alkynyl secondary carbon

C4a	R-CH=O / aldehyde carbon

C5a	R-C(=O)-R / keto carbon
C5x	ring-C(=O)-ring / keto carbon in ring

C6a	R-C(=O)-OH / carboxylate carbon

C7a	R-C(=O)-O-R / carboxylate ester carbon
C7x	ring-C(=O)-O-ring / lactone carbon

C8x	ring-CH=ring / aromatic secondary carbon
C8y	ring-C(-R)=ring / aromatic tertiary carbon

C0	Undefined carbon

	**Nitrogen atoms**

N1a	R-NH2 / primary amine
N1b	R-NH-R / secondary amine
N1c	R-N(-R)2 / tertiary amine
N1d	R-N(-R)3+ / quaternary ammonium
N1x	ring-NH-ring / secondary amine in ring
N1y	ring-N(-R)-ring / tertiary amine in ring

N2a	R=N-H / primary imine
N2b	R=N-R / secondary imine
N2x	ring-N=ring / secondary imine in ring
N2y	ring-N(-R)+=ring / iminium

N3a	R#N / nitrile

N4x	ring-NH-ring / aromatic secondary amine
N4y	ring-N(-R)-ring / aromatic tertiary amine

N5x	ring-N=ring / aromatic secondary imine

N5y	ring-N(-R)+=ring / aromatic tertiary imine

N0	Undefined nitrogen

	**Oxygen atoms**

O1a	R-OH / hydroxy
O1b	N-OH / N-hydroxy
O1c	P-OH / P-hydroxy
O1d	S-OH / S-hydroxy

O2a	R-O-R / hydroxy ether
O2b	P-O-R / hydroxy phosphate bond
O2c	P-O-P / pyrophosphate bond
O2x	ring-O-ring / cyclic ether

O3a	N=O / N-oxo
O3b	P=O / P-oxo
O3c	S=O / S-oxo

O4a	R-CH=O / aldehyde oxygen

O5a	R-C(=O)-R / keto oxygen
O5x	ring-C(=O)-ring / keto oxygen in ring

O6a	R-C(=O)-OH / carboxylate oxygen

O7a	R-C(=O)-O-R / carboxylate ester oxygen
O7x	ring-C(=O)-O-ring / lactone oxygen
O0	Undefined oxygen

	**Sulfur atoms**

S1a	R-SH / mercapto

S2a	R-S-R / sulfide
S2x	ring-S-ring / sulfide in ring

S3a	R-S-S-R / disulfide
S3x	ring-S-S-ring / disulfide in ring

S4a	R-SO3 / sulfate
S0	Undefined sulfur

	**Phosphorus atoms**

P1a	P-R / phosphine
P1b	P-O / phosphate

	**Halogen atoms**

X	F / fluoride
	Cl / chloride
	Br / bromide
	I / iodide

	**Other atoms**

Z	Other atoms

KEGG Atom Types were defined in 2003 [17], and were used to label the nodes in molecular graphs. KEGG atom label consists of three letters, such as "C1a" meaning a methyl carbon. The first and second letters represent atom species and orbital environments, respectively. The third letter describes the surroundings of a given atom in terms of its bonded neighbors.

### Reactant pairs and compound pairs

A reactant pair is part of a reaction equation, representing a set of substrate and product with conserved chemical moiety [19]. KEGG RPAIR database defines 14,105 reactant pairs as of June 2013. In this study, we used the reactant pairs with "main" types, representing the main flow of atoms, as the positive examples of the *de novo *metabolic pathway reconstruction.

The possible combinations of compound pairs, other than the ones defined as reactant pairs, are used as negative examples. 6,922 compounds were involved in known reactions, therefore, distinguishing the two distinct directions, *i.e*., forward and backward, the number of all the compound pairs was 47,907,162.

### Conventional chemical fingerprints

We used conventional chemical fingerprints in order to compare the KCF-S descriptors (explained in the Method section) for the interpretability of characterising molecule datasets and for the predictive ability of *de novo *pathway reconstruction. Chemical fingerprints encode presence or absence (1 or 0) of chemical substructures in molecules, resulting in a high dimensional binary vector. We used the Chemistry Development Kit (CDK) version 1.4.9 [16] to calculate well-known fingerprints, MACCS fingerprint and PubChem fingerprint. Their dimensions are 164, and 879, respectively.

## Methods

In this section, we present a novel integer vector representation of chemical compound named "KCF-S descriptor", each element of which corresponds to the number of a substructure included in a chemical compound. We define such substructures on biochemist's notion of substructures of a chemical compound. We also make a brief review of methods for compound clustering and metabolic pathway reconstruction to show the applicability of the KCF-S descriptor in the Results and Discussion section.

### Proposed definition of biochemical substructures in KCF-S

Every biochemical substructure was computationally represented as a graph object, with non-hydrogen atoms and bonds described as nodes and edges, respectively, as an extension of the method in Kotera *et al *[2]. They were computationally defined using seven attributes: ATOM, BOND, TRIPLET, VICINITY, RING, SKELETON, and INORGANIC. In this study, each substructure was given a label (string of characters) using KEGG Atom Types so that the substructures can be distinguished to each other and be interpreted by the words in organic chemistry or biochemistry.

**Figure 2:**
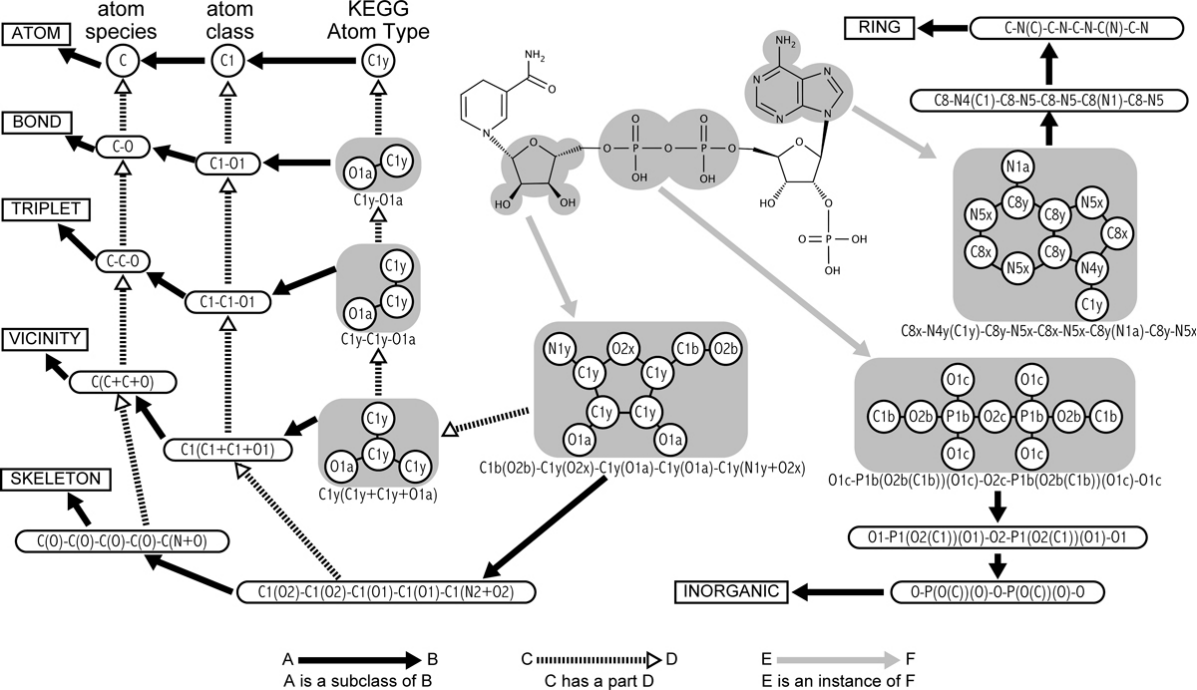
**Examples of proposed KCF-Substructures and their relationships**. Three types of arrows are used for explaining the relationships between objects. See the text for the detail.

Figure 2 shows example substructures obtained from NADH. In this figure, the graph objects in gray areas represents examples of substructures defined in this study. For example, around the center of Figure 2, there is a substructure labeled as "C1b(O2b)-C1y(O2x)-C1y(O1a)-C1y(O1a)-C1y(N1y+O2x)", which is one of the SKELETON entries extracted from a molecule NADH. This SKELETON entry represents a ribose residue in this molecule. In other words, a ribose residue is an instance of a substructure "C1b(O2b)-C1y(O2x)-C1y(O1a)-C1y(O1a)-C1y(N1y+O2x)", which is a subclass of SKELETON. These "instance_of" and "subclass_of" relationships are described by gray and black arrows in Figure 2, respectively. Note that an atom (or a node) can belong to more than one substructure entries. For example, one of the furanose forms of ribose residues in NADH contains a furan ring (five-membered ring consisting of four carbon and one oxygen atoms) that is a subclass of RING. Similarly, sugar residues (*e.g*., ribose residue) contain many secondary hydroxyl groups that are represented as a subclass of BOND. In other words, a furanose form of ribose residue has a furan ring, and a sugar residue has secondary hydroxyl groups. This "has_part" relationships are described by dotted arrows in Figure 2. The definitions of the seven attributes of substructures are explained below.

#### The ATOM attribute in KCF-S

An ATOM entry represents KEGG Atom Type (Table 1). In Figure 2, circles represent ATOM entries, corresponding to the nodes that form molecular graphs. For example, "C1y" in Figure 2 is one of such nodes. According to the definition of the KEGG Atom Types, the ATOM entries were classified hierarchically (described by black solid arrows). A KEGG Atom Type (*e.g*., "C1y") is a subclass of the atom classes represented by the first two letters (termed as KEGG Atom Classes, *e.g*., "C1"). A KEGG Atom Class (*e.g*., "C1") is a subclass of the element (*e.g*. carbon atom), which is a subclass of ATOM entries. In KCF-S, the variable *k *represents the level of the ATOM attributes: *k1, k2 *and *k3 *mean the atom species (or elements), atom classes and atom types, respectively.

#### The BOND attribute in KCF-S

A BOND entry is defined as a pair of ATOM entries that form a chemical bond in a molecule, corresponding to many named bonds in organic chemistry and biochemistry (*e.g*., C5a-S2a for carboxylic thioester bond). In Figure 2, the substructure labeled as "C1y-O1a" is shown as an example of a BOND entry, which represents a secondary hydroxyl group on a cyclic structure. In the string that identifies a BOND entry, two ATOM entries were sorted in the alphabetical order, and were connected with a hyphen. This BOND entry was classified according to the hierarchy defined for the ATOM entries; *i.e*., "C1y-O1a" bond is a subclass of "C1-O1" bond, "C1-O1" bond is a subclass of "C-O" bond, and "C-O" bond is a subclass of a BOND (described by black arrows). Also, a BOND entry has two ATOM entries, and a BOND is part of many other entries (as described by dotted arrows).

#### The TRIPLET attribute in KCF-S

A TRIPLET entry is defined as a pair of BOND entries that share a central ATOM, which consistis of three ATOMs that are connected sequentially. For example, the triplet "C6a-C1c-N1a", "C6a-C1c-O1a" and "C6a-C5a-O5a" represent the common substructures in alpha-amino acids, alpha-hydroxy acids and alpha-oxo acids, respectively. In the string that identifies a TRIPLET entry, the BOND entries were sorted in the alphabetical order of the ATOM entries, three ATOM entries were connected with hyphens so that the central ATOM was placed in the middile. In Figure 2, the triplet "C1y-C1y-O1a" is shown as an example of TRIPLET entries, which represents a larger substructure that contains a secondary hydroxyl group on a cyclic structure. Similary with the BOND entries, the TRIPLET entries were classified according to the hierarchy defined for the ATOM entries. A TRIPLET entry has two BOND entries and three ATOM entries, and TRIPLET is part of many other entries.

#### The VICINITY attribute in KCF-S

A VICINITY entry is defined as a central atom and the atoms attached to it. Many functional groups correspond to VICINITY entries, *e.g*., carbamate "C7a(O6a+O7a+N1b)", N-acetyl "C5a(C1a+N1b+O5a)", and phosphate "P1b(O1c+O1c+O1c+O2b)". In Figure 2, the vicinity "C1y(C1y+C1y+O1a)" is shown as an example, which represents an even larger substructure that contains a secondary hydroxyl group on a cyclic structure. In the string that identifies a VICINITY entry, the central ATOM was placed in the head, and the attaching ATOM entries were sorted in the alphabetical order, connected with plus signs, and placed in parentheses. A VICINITY entry consists of at least three BOND entries and at least four ATOM entries.

#### The RING attribute in KCF-S

A RING entry is defined as a cyclic substructure, containing 3-, 4-, 5- and 6-membered, or larger (up to 12-membered), rings. The strings to identify RING entries were generated in the following way: (i) an atom in the ring was selected as a starter to retrieve ring structures using depth-first search algorithm, (ii) KEGG Atom Types consisting of the ring were connected by hyphens to generate a backbone string, (iii) if there were branch atoms attached to the ring, they were added to the backbone string using parentheses, (iv) the processes (i)-(iii) were repeated for all starting atoms, clockwise and anti-clockwise directions, (v) the obtained strings were sorted in alphabetical order, and (vi) the first string was selected to represent the RING entry.

Some common examples are the phenyl ring "C8x-C8x-C8x-C8x-C8x-C8y(C1b)", imidazole ring "C8x-C8y(C1b)-N5x-C8x-N4x" and pyrrole ring "C8x-C8x-C8y(C1b)-C8x-N4x". Pyranose sugar ring was represented as "C1y(C1b)-C1y(O1a)-C1y(O1a)-C1y(O1a)-C1y(O2a)-O2x".

RING also deals with condensed rings. For example, adenine in NADH was represented as a 9-membered condensed ring "C8x-N4y(C1y)-C8y-N5x-C8x-N5x-C8y(N1a)-C8y-N5x", consisting of a 5-membered ring "C8x-N4y(C1y)-C8y-C8y-N5x" and a 6-membered ring "C8x-N5x-C8y(N1a)-C8y-C8y-N5x".

#### The SKELETON attribute in KCF-S

A SKELETON entry is defined as a carbon skeleton/backbone, such as alkyl and aryl groups. The strings to identify SKELETON entries were generated in the following way: (i) a carbon atom in the terminus of the carbon skeleton was selected as a starter to retrieve all carbon chains in the skeleton, (ii) KEGG Atom Types consisting of the chains were connected by hyphens, (iii) if other elements (N, O, S, *etc*) attach to the chain, they were added to the chain using parentheses, (iv) the longest chain was selected as a seed, and the shorter chains were bundled to generate the string representing the carbon skeleton, (v) the processes (i)-(iv) were repeated for all starting atoms, (vi) the obtained strings were sorted in alphabetical order, and

(vii) the first string was selected to represent the SKELETON entry.

Some common examples are the N-acetyl group "C1a-C5a(O5a+N1b)", O-acetyl group "C1a-C7a(O6a+O7a)", and hexopyranose sugar ring O-glycoside "C1b(O1a)-C1y(O2x)-C1y(O1a)-C1y(O1a)-C1y(O1a)-C1y(O2a+O2x)".

#### The INORGANIC attribute in KCF-S

An INORGANIC entry is defined as a connected atom groups that consists of elements that are not carbon atoms. The strings to identify INORGANIC entries were generated in the following way: (i) an atom in the terminus of the inorganic component was selected as a starter to retrieve all chains in the inorganic component, (ii) KEGG Atom Types consisting of the chains were connected by hyphens, (iii) if carbon atoms attach to the chain, they were added to the chain using parentheses, (iv) the longest chain was selected as a seed, and the shorter chains were bundled to generate the string representing the inorganic component, (v) the processes (i)-(iv) were repeated for all starting atoms, (vi) the obtained strings were sorted in alphabetical order, and (vii) the first string was selected to represent the INORGANIC entry.

Some common examples are primary alcohol phosphate ester "O1c-P1b(O2b(C1b))(O1c)-O1c", and sulfonate "O1d-S4a(C1b)(O1d)-O1d".

### Compound clustering based on the KCF-S descriptors

We perform a hierarchical agglomerative clustering of compounds described by the KCF-S descriptors using a variant of quasi-clique-based clustering (QCC), which was originally developed for clustering of large amount of genes to detect orthologs in KEGG OC [20].

In the original QCC algorithm, each object is represented by a neighbor profile in which each element corresponds to a similarity score with the other objects, and the object-object similarity is evaluated by the inner product of the neighbor profiles. The key parameter of the QCC algorithm is the clique ratio that decides whether or not two clusters should be connected. For example, when the clique ratio is set to 1.0, two clusters should be connected if the similarity scores of all object pairs in the clusters are above the similarity threshold. In this case, this QCC method is equivalent to complete-linkage clustering. When the clique ratio is below 1.0, *e.g*., 0.7, two clusters should be connected if 70% of the object pairs in the clusters are above the similarity threshold.

In this study, instead of the inner product of the neighbor profiles in the original QCC, we used the weighted Jaccard coefficient of the KCF-S descriptors. We also make a comparison of the clustering result between the KCF-S descriptors and conventional fingerprints (*e.g*., PubChem/MACCS fingerprints).

### Metabolic pathway reconstruction based on the KCF-S descriptors

Our previous study for the *de novo *metabolic pathway reconstruction [15] predicts a series of reactions of each pair of chemical compounds on a metabolic pathway by solving the following supervised classification problem. Given a collection of *n*(*n−*1) compound-compound pairs (*C_i _, C_j_*)(*i *= 1 , . . . , *n, j *= 1, . . . , *n, i ≠ j*), we estimate a linear function *f*(*C, C'*) that would predict whether or not a chemical compound *C *is converted to another compound *C' *in an enzymatic reaction.

Linear models use feature vectors for predictions. Our feature vectors are a generalization of the previous ones [15] from binary vectors to integer vectors. Our KCF-S descriptor represents compounds *C *and *C' *as *D*-dimensional integer vectors as Φ(*C*) = (*c*_1_, *c*_2_, . . . , *c_D_*)*^T ^*and $\text{Φ}\left(C^{\prime }\right)\;=\;{\left({c^{\prime }}_{1},\;{c^{\prime }}_{2},\;.\;.\;.\;,\;{c^{\prime }}_{D}\right)}^{T}$, respectively, where *c_k_*, $c_{k}^{\prime }\in \mathbb{Z},k=\text{ 1},\ldots \;,D$. Let min(*c_k_*, $c_{k}^{\prime }$) be a function that returns *c_k _*if $c_{k}\leq c_{k}^{\prime }$ and otherwise returns $c_{k}^{\prime }$, and let max(*c_k_*, $c_{k}^{\prime }$) be a function that returns *c_k _*if $c_{k}\leq c_{k}^{\prime }$ and otherwise returns $c_{k}^{\prime }$. We define two operations for the descriptors as follows:

$$\left(\text{Φ}\left(C\right)\wedge \text{Φ}\left(C^{\prime }\right)\right)\;=\left(\text{min}\left(c_{\text{1}},c_{1}^{\prime }\right),\;\text{min}\left(c_{\text{2}},\;c_{2}^{\prime }\right),.\;.\;.\;,\text{min}\left(c_{n},c_{n}^{\prime }\right)\right)$$

and

$$(\Phi \left(C\right)⊖\Phi (C^{\prime }))\;=(\max(c_{\text{1}}-c_{1}{}^{\prime },0),\;\max(c_{\text{2}}-\;c_{2}{}^{\prime },0),.\;.\;\;.\;,\text{max}(c_{n}-c_{n}{}^{\prime },0)).$$

The both operations are generalizations of the previously defined operations [15] from binary vectors to integer vectors. (Φ(*C*) ∧ Φ(*C'*)) captures common KCF-S features between Φ(*C*) and Φ(*C'*), while $\left(\text{Φ}\left(C\right)⊖\text{Φ}\left(C^{\prime }\right)\right)$ captures KCF-S features present in Φ(*C*) and absent in Φ(*C'*). (Φ(*C*) ∧ Φ(*C'*)) and $\left(\text{Φ}\left(C\right)⊖\text{Φ}\left(C^{\prime }\right)\right)$ are referred to as common features and differential features, respectively. Using the above operations, we represent any compound-compound pair by two types of feature vectors as follows:

$$\text{Φ}\left(C,C^{\prime }\right)={\left(\text{Φ}\left(C\right)\wedge \text{Φ}\left(C^{\prime }\right),\text{Φ}\left(C\right)\;⊖\;\text{Φ}\left(C^{\prime }\right),\text{Φ}\left(C^{\prime }\right)\;⊖\;\text{Φ}\left(C\right)\right)}^{T}$$

and

$$\bar{\text{Φ}\left(C,C^{\prime }\right)}={\left(\text{Φ}\left(C\right)\;⊖\;\text{Φ}\left(C^{\prime }\right),\text{Φ}\left(C^{\prime }\right)\;⊖\;\text{Φ}\left(C\right)\right)}^{T}.$$

The both feature vectors are also generalizations of the previously defined feature vectors [15]. Φ(*C, C'*) and $\bar{\text{Φ}\left(C,C^{\prime }\right)}$ are referred to as "diff-common feature vector" and "diff-only feature vector", respectively. Note that the diff-common and diff-only feature vectors share the differential features, but the diff-common feature vector additionally has the common features. Thus, the both feature vectors are designed to capture substructure changes around the reaction center in the conversion of a chemical compound to another compound. In addition, the diff-common feature vector is designed to capture core substructures kept in the conversion of a chemical compound to another compound.

Using the feature vectors Φ(*C, C*') and $\bar{\text{Φ}\left(C,C^{\prime }\right)}$ for compounds *C *and *C'*, a linear model estimates a linear function *f*(*C, C'*) = **w***^T^*Φ(*C, C'*), where **w **is a real value vector (weight vector). The reaction between *C *and *C' *is predicted by thresholding the value of *f*(*C, C'*). The weight vector **w **is estimated such that it can predict enzymatic-reaction likeness of compound-compound pairs. To estimate the weight vector **w**, we apply linear support vector machine (SVM) with *L*_1_-regularization for its high interpretability and high prediction accuracies comparable to SVM with *L*_2_-regularization. To solve the optimization problem in SVM, we use an efficient optimization algorithm named LIBLINEAR [21], which is available from http://www.csie.ntu.edu.tw/~cjlin/liblinear/.

## Results and discussion

### KCF-S (KEGG Chemical Function and Substructure) format

**Figure 3:**
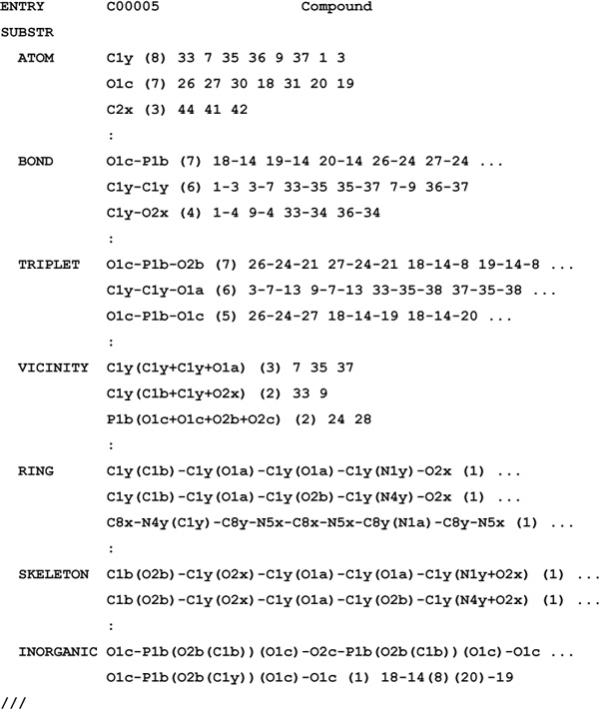
**KEGG Chemical Function and Substructures (KCF-S) format, a proposed extension of KCF ** KCF-S format has two sections, ENTRY and SUBSTR (substructures). SUBSTR section is divided into the seven subsections, ATOM, BOND, TRIPLET, VICINITY, RING, SKELETON and INORGANIC. Each subsections contains the substructures with the strings, the number of the substructures appeared in the molecule (shown in the parentheses), and the atoms involved in the substructures.

Figure 3 represents an example of KCF-S format proposed in this study, where the seven attributes (ATOM, BOND, TRIPLET, VICINITY, RING, SKELETON and INORGANIC) are listed with the KEGG Atom strings, appearances in the molecule, and the atoms involved in the substructures.

KCF format of molecules have been provided in KEGG as a fundamental chemical structure information since 2003 [17]. The aim of developing another format named KCF-S format is not to replace KCF into KCF-S, but to provide additional information of larger substructures for the correspondence with the names in organic chemistry and biochemistry, and for the application for many analyses such as structure-based clustering of molecules and metabolic pathway reconstruction study. Note that both of KCF and KCF-S formats can be automatically converted from Molfile format. This means that, even though we only used molecules in KEGG and KNApSAcK databases in this study, KCF and KCF-S can deal with many other molecules in PubChem [22], ChEBI [23], DrugBank [24], NCI [25] and other databases.

### Appearances of substructures in the KEGG and KNApSAcK databases

The three databases collect molecules for different purposes, *i.e*., KEGG COMPOUND for fundamental biological systems, KEGG DRUG for pharmaceuticals, and KNApSAcK for secondary metabolites. Therefore, even though they share some molecules, their collection of molecules are different from each other. The appearance of substructures made it possible to grasp more detailed characteristics of their databases.

#### Examples of named substructures

Table 2 shows examples of named substructures and appearance in KEGG COMPOUND, KEGG DRUG and KNApSAcK databases. These three databases have been collecting molecules in different purposes, so the appearance of the substructures is naturally different, which is clearly shown in this study. For example, BOND entries include many named bonds such as amide bond "C5a-N1b" and carboxylate ester bond "C7a-O7a". About 13% of molecules (2,192 molecules) in KEGG COMPOUND have amide bonds labeled as "C5a-N1b", and the number of the bond in total was 4,174 (about 1.9 bonds per molecule). About 5% of molecules (2,528 molecules) in KNApSAcK have the same bond, and the number of the bond in total was 6,784 (about 2.7 bonds per molecule). This means that, even though KNApSAcK contains about three times more molecules than KEGG COMPOUND, proportion of molecules containing the bonds in KNApSAcK is not as high as that of KEGG COMPOUND, but the average number of the bonds is larger in KNApSAcK.

**Table 2:** Examples of named substructures and appearance in KEGG COMPOUND, KEGG DRUG and KNApSAcK databases.

KCF-S / annotation	COMPOUND	DRUG	KNApSAcK
	#S / #C	#S / #C	#S / #C
BOND			
C5a-N1b / amide bond	4174 / 2192	2678 / 1385	6784 / 2528
C7a-O7a / carboxylate ester bond	3040 / 2198	1787 / 1329	21857 / 13166
C5a-S2a / thioester bond	455 / 453	31 / 30	36 / 36
N2b-N2b / diazo bond	83 / 73	83 / 19	11 / 11
S3a-S3a / disulfide bond	40 / 37	40 / 26	43 / 33
N1b-N1b / hydrazine bond	15 / 13	22 / 15	3 / 3

TRIPLET			
C6a-C1c-N1a / alpha-amino acid	512 / 484	113 / 104	191 / 183
C5a-C1b-C5a / beta-keto carbonyl	270 / 106	6 / 6	36 / 36
C6a-C5a-O5a / alpha-keto carboxylate	169 / 168	10 / 8	46 / 46
C6a-C1c-O1a / alpha-hydroxy carboxylate	167 / 154	236 / 137	108 / 87

VICINITY			
C1y(C1y+C1y+O1a) / cyclic secondary alcohol	10099 / 3090	1171 / 388	49015 / 11697
C8y(C8x+C8x+O1a) / phenolic hydroxy	1562 / 1263	376 / 313	9978 / 7219
C5a(N1b+N1b+O5a) / pseudourea	66 / 65	82 / 77	46 / 43
N1c(C1b+C1b+C1b) / tertiary amine	54 / 48	302 / 235	0 / 0
C5x(N1x+N1x+O5x) / cyclic pseudourea	36 / 36	30 / 29	20 / 20

RING			
C1y(C1b)-C1y(O1a)-C1y(O1a)-C1y(O1a)-C1y(O2a)-O2x / pyranose sugar ring	1024 / 824	64 / 54	7670 / 6187
C8x-N4y(C1y)-C8y(N5x)-C8y(C8y)-N5x / imidazole ring	549 / 535	48 / 47	84 / 84
C8x-N4y(C1y)-C8y-N5x-C8x-N5x-C8y(N1a)-C8y-N5x / adenine ring	428 / 420	17 / 17	55 / 55
C1x-C1x-N1y(C1b)-C1x-C1x-N1y(C1b) / piperazine ring	7 / 7	45 / 45	0 / 0
C8x-C8y(C2b)-C8x-C8y(O1a)-C8y(O1a)-C8y(O1a) / 5-alenylbenzene-1,2,3-triol	3 / 3	0 / 0	12 / 12

SKELETON			
C1b(O2b)-C1y(O2x)-C1y(O1a)-C1y(O1a)-C1y(N4y+O2x) / ribofuranose	255 / 255	20 / 20	62 / 62
C1x(N1y)-C1x(N1y) / ethylenediamine in ring	136 / 136	702 / 702	0 / 0
C1a-C1c(C1a)-C1b-C1c(N1b)-C5a(N1b+O5a) / leucine residue	102 / 102	79 / 79	228 / 228
C7a(O6a+O7a)-C8y-C8x-C8x-C8y(O2a)-C8x-C8x / p-hydroxybenzoate residue	0 / 0	3 / 3	51 / 51

INORGANIC			
O1c-P1b(O2b(C1y))(O1c)-O1c	520 / 520	19 / 19	66 / 66
/ cyclic secondary alcohol orthophosphate			
O1c-P1b(O2b(C1b))(O1c)-O1c	387 / 387	43 / 43	97 / 97
/ primary alcohol orthophosphate			
O1c-P1b(O2b(C1y))(O2b(C1b))-O1c / cyclic orthophosphate	173 / 173	2 / 2	2 / 2
O3a-N2b(C8y)-O3a / aryl nitro	304 / 304	164 / 164	48 / 48
N2b(C2c)-O1b / oxime	27 / 27	22 / 22	61 / 61

#S represents the numbers of KCF-Substructures, and #C represent the numbers of compounds containing the KCF-Substructures. Note that the annotations are not necessary-and-sufficient definitions. For example, "N1b-N1b" bond is a hydrazine bond, but there are some other types of hydrazine bonds; e.g., "N1b-N1c" is a hydrazine bond with three substituted groups, and "N1x-N1x" is a hydrazine bond in a ring structure.

In contrast, about 14% (2,198) molecules in COMPOUND have "C7a-O7a" carboxylate ester bond, whereas about 26% (13,166) molecules in KNApSAcK have the same bond.

In addition to essential amino acids, there are many other alpha-amino acids. the TRIPLET attribute grasps the substructure that defines alpha-amino acids "C6a-C1c-N1a", which resulted in finding 484 (2.8%) molecules in COMPOUND, 104 (1.0%) molecules in DRUG, and 183 (0.36%) molecules in KNApSAcK.

VICINITY entries define more detailed substructures. For example, the atom class "O1" sufficiently describe a hydroxy group (see Table 1). Among these, the KEGG Atom "O1a" describe a hydroxy group attached to a carbon atom, which is usually referred to as an alcohol group. It is known that primary alcohol group, secondary alcohol group and tertiary alcohol group are different in terms of the reactivity in organic chemistry, and they are distinguished by the BOND entries "C1b-O1a", "C1c-O1a" and "C1d-O1a", respectively. Secondary and tertiary alcohols can be in a ring structure, cyclic secondary alcohol and cyclic tertiary alcohol, and in such cases they are represented as the BOND entries "C1y-O1a" and "C1z-O1a", respectively. The VICINITY entry "C1y(C1y+C1y+O1a)" defines even more detailed subclass of cyclic secondary alcohol, and sugar residues contain many of these entries. Similarly, the BOND entry "C8y-O1a" sufficiently describe a phenolic hydroxy group, and the VICINITY entry "C8y(C8x+C8x+O1a)" defines the phenolic hydroxy group that does not have any substituted groups in the ortho (o-) positions.

RING, SKELETON, and INORGANIC entries captured many substructures that have been defined in literatures in organic chemistry and biochemistry but have not been usually captured by the conventional chemical fingerprints. For example, an RING entry "C8x-N4y(C1y)-C8y-N5x-C8x-N5x-C8y(N1a)-C8y-N5x" represented an adenine ring that is attached with a carbon atom in a ring structure (usually a ribose residue). This adenine ring and the imidazole ring "C8x-N4y(C1y)-C8y(N5x)-C8y(C8y)-N5x" are examples of the RING entries that are frequently found in COMPOUND but not in DRUG and KNApSAcK databases. In contrast, piperazine ring "C1x-C1x-N1y(C1b)-C1x-C1x-N1y(C1b)" is an example RING entry that are frequently found only in DRUG database.

Many sugar rings were found in RING entries, including a pyranose sugar ring "C1y(C1b)-C1y(O1a)-C1y(O1a)-C1y(O1a)-C1y(O2a)-O2x". Sugar residues were also found in SKELETON entries, such as a ribofuranose "C1b(O2b)-C1y(O2x)-C1y(O1a)-C1y(O1a)-C1y(N4y+O2x)" that is attached to an aromatic nitrogenous ring such as nucleic bases. SKELETON entries captured many named amino acid residues such as leucine residue" C1a-C1c(C1a)-C1b-C1c(N1b)-C5a(N1b+O5a)".

INORGANIC entries contained orthophosphate, pyrophosphate, sulfate, sulfite, nitro, *etc*, and the variations and the positions of substituted groups were discriminated, such as primary alcohol orthophosphate "O1c-P1b(O2b(C1b))(O1c)-O1c", cyclic secondary alcohol orthophosphate "O1c-P1b(O2b(C1y))(O1c)-O1c" and cyclic orthophosphate "O1c-P1b(O2b(C1y))(O2b(C1b))-O1c".

#### Statistics of the substructures in KEGG and KNApSAcK databases

**Figure 4:**
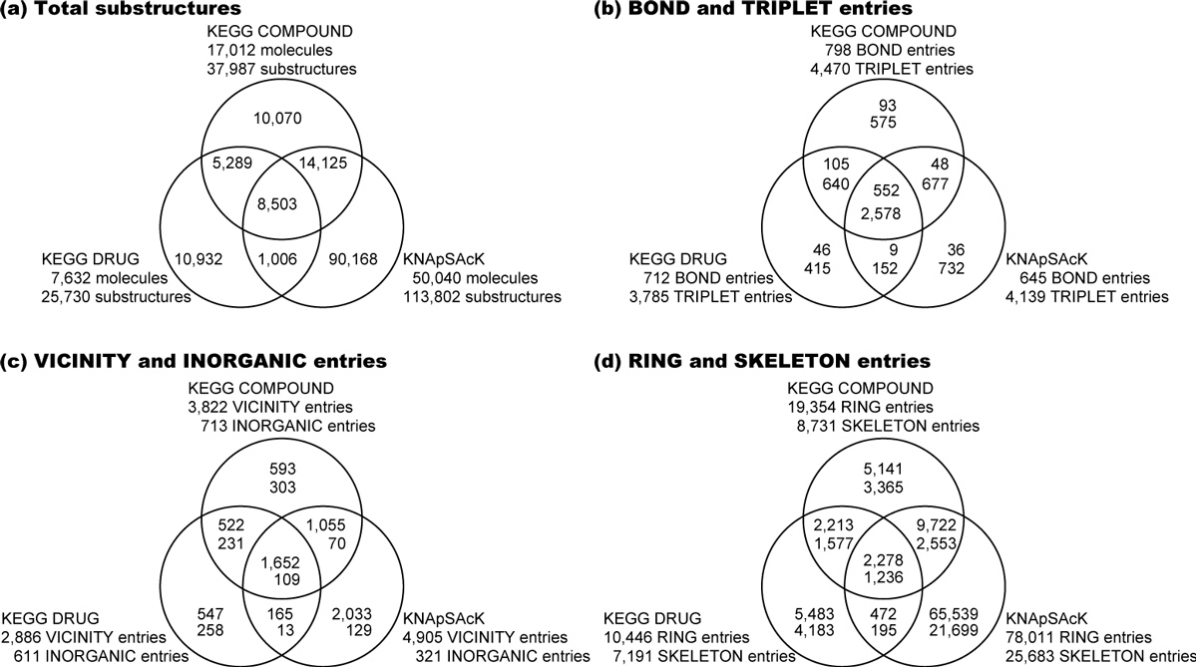
**Venn diagrams for common and uniq KCF Substructures in the KEGG COMPOUND, KEGG DRUG, KNApSAcK databases**. The numbers of (a) KCF-Substructures, (b) BOND and TRIPLET entries, (c) VICINITY and INORGANIC entries, and (d) RING and SKELETON entries are shown in the top and bottom, respectively.

The numbers of KCF Substructures obtained from the KEGG COMPOUND, KEGG DRUG and KNApSAcK databases were summerized in Figure 4. From the three databases, 140,093 substructures were obtained, among which only 8,503 (6.1%) appeared in all of the three databases. Among the 37,987 substructures from KEGG COMPOUND, 10,070 substructures (27%) were unique (not found in other databases). Similarly, among the 25,730 and 113,802 substructures from KEGG DRUG and KNApSAcK databases, 10,932 (42%) and 90,168 (79%) substructues were unique, respectively (Figure 4a).

Each of the 68 KEGG Atom Types consists of 1-3 characters that hierarchically classify microenvironment of atoms. For example, carbon atoms "C" are classified into alkyl carbon atoms "C1", alkenyl carbon atoms "C2", *etc*., and alkyl carbon atoms "C1" are further classified into "C1a", "C1b", *etc*. (see Table 1), which comes up to 98 ATOM entries. All of the three databases use all these ATOM entries.

From the three databases, 889 BOND entries were obtained, among which 552 (62%) appeared in all of the three databases (Figure 4b). Among the 798 BOND entries from KEGG COMPOUND, 93 substructures (12%) were unique (not found in other databases). Similarly, among the 712 and 645 BOND entries from KEGG DRUG and KNApSAcK databases, 46 (6.5%) and 36 (5.6%) substructues were unique, respectively.

5769 TRIPLET entries were obtained in total from the three databases, among which 2578 (45%) were shared (Figure 4b). Unique TRIPLET entries in KEGG COMPOUND, KEGG DRUG and KNApSAcK databases were 575 out of 4,470 (13%), 415 out of 3,785 (11%) and 732 out of 4,139 (18%), respectively.

From the 6567 VICINITY entries obtained in total, only 1,652 (25%) were shared in the all three databases (Figure 4c). 593 out of 3,822 (16%) and 547 out of 2,886 (19%) were unique in KEGG COMPOUND and KEGG DRUG, respectively, whereas it was found that KNApSAcK database had as many as 2,033 out of 4,905 (41%) unique VICINITY entries.

The proportion of the shared entries were even fewer in INORGANIC, RING and SKELETON entries, which were 109 out of 1,113 (9.8%), 2,278 out of 90,848 (2.5%), and 1,236 out of 34,808 (3.6%), respectively (Figure 4c and 4d). The numbers of unique entries in KEGG COMPOUND were generally small; 303 (42%) out of 713 INORGANIC entries, 5,141 (27%) out of 19,354 RING entries, and 3,365 (39%) out of 8,731 SKELETON entries. Those in KEGG DRUG were larger; 258 (42%) out of 611 INORGANIC entries, 5,483 (52%) out of 10,446 RING entries, and 4,183 (58%) out of 7,191 SKELETON entries. KNApSAcK generally had even more entries; 129 (40%) out of 321 INORGANIC entries, 65,539 (84%) out of 78,011 RING entries, and 21,699 (84%) out of 25,683 SKELETON entries.

#### Characteristic appearance of substructures in respective datasets

We further investigated the characteristic appearance of substructures in respective databases in the following way: the numbers of molecules that do or do not contain the respective substructures are counted in a database and another, and the significantly appearing substructures in the database against those in the other were ranked according to the P-value using Fisher's exact test. The top five characteristic substructures in respective attributes are shown in the Supplementary tables S1-S6 in Additional file 1.

By the comparison of KEGG COMPOUND with KEGG DRUG, it was shown that KEGG COMPOUND has significantly more molecules that contain sugar residues, phosphate groups and adenine residues (Table S1), which reflects that KEGG COMPOUND collects molecules related with fundamental biological systems such as nucleic acids and sugar phosphates.

Comparing KEGG DRUG with KEGG COMPOUND, secondary and tertiary amines, aromatic rings, aryl halides, piperazine rings, ethylenediamine and ethanolamine residues, and sulfur-related inorganic residues were found to be characteristic in KEGG DRUG (Table S2). Similarly, comparison of KNApSAcK with KEGG COMPOUND revealed that carboxylate ester bonds, especially alkyl carboxylate ester bonds, and O-acetyl group was found to be characteristic in KNApSAcK (Table S3). These comparisons reflects the nature of molecules in the respective databases, *i.e*., DRUG for pharmaceuticals and KNApSAcK for secondary metabolites.

The same analysis can be conducted using any datasets of molecules. In other words, as demonstrated above, the KCF-S enables us to find characteristic substructures in any given datasets of molecules in a way that the obtained substructures are interpretable with the words in biochemistry and organic chemistry.

### Structure-based clustering of molecules using KCF-S descriptors

As the first application of KCF-S, we conducted the structure-based clustering of the molecules in the following way; the structures of molecules were represented in the form of the KCF-S descriptors (integer vectors), the similarity between the molecules were defined as a weighted Jaccard coefficient between the two corresponding KCF-S descriptors, and the complete-linkage clustering or the QCC methods were applied with a variety of thresholds.

Table 3 shows the comparison of the five complete-linkage clusters with weighted Jaccard coefficient *>*= 0.7 using KCF-S and PubChem/MACCS fingerprints. It appeared that PubChem and MACCS fingerprints tend to generate larger clusters than those by KCF-S. KCF-S generated more numbers of smaller clusters, and the clusters generally consisted of the molecules sharing the same core structures. It was also observed that the clusters obtained by PubChem and MACCS fingerprints do not take into account the number of substituted groups, such that the standard deviation of the molecular weights were generally larger than the clusters obtained by KCF-S descriptor. Many clusters obtained by KCF-S descriptor can be described by the names of compound classes, such as acyl-CoA and disaccharides. In contrast, many clusters obtained by PubChem and MACCS fingerprints were so diverse that we could not find appropriate words to describe the clusters.

**Table 3:** Top five complete-linkage clusters with weighted Jaccard coefficient >= 0.7.

(a) clustered by KCF-S descriptor								
**Cluster**	**#M**	**Max MW**		**Ave MW**		**Min MW**		**SD**

#1 acyl-CoA molecules								
	144	993.8	C01894	883.8	C04348	767.5	C00010	3.317

#2 enoyl-CoA molecules								
	79	1124	C16388	1026	C16163	891.7	C05276	6.789

#3 metals and inorganic ions								
	48	244.0	C19159	97.75	C00150	1.00	C00080	10.11

#4 acyl-CoA molecules with aromatic substituted groups								
	48	1023	C14118	929.6	C00323	861.6	C00845	6.107

#5 disaccharides								
	35	342.2	C00897	339.3	C04698	326.2	C19758	1.153

(b) clustered by PubChem fingerprint								

**Cluster**	**Molecules**	**Max MW**		**Ave MW**		**Min MW**		**SD**

#1 from furanocoumarins to glycosylated flavonoids								
	382	918.8	C12636	372.7	C09956	186.1	C09060	5.993

#2 from biotinyl-5'-AMP to CoA-disulfide								
	237	1533	C02015	959.5	C16339	573.5	C05921	7.893

#3 from flavonoids to pyrones (chromones), aggregated phenols								
	159	668.7	C10669	325.1	C09752	166.1	C10712	6.879

#4 from xanthenes to tannins, glycosylated and acylated flavonoids								
	156	2108	C16302	757.2	C12646	346.2	C09967	27.82

#5 steroids								
	135	514.2	C15359	335.8	C14621	270.3	C14261	3.703

(c) clustered by MACCS fingerprint								

**Cluster**	**Molecules**	**Max MW**		**Ave MW**		**Min MW**		**SD**

#1 from pyrimidine 5'-deoxynucleotide to CoA-disulfide								
	432	1533	C02015	823.4	C00100	277.1	C08249	12.13

#2 from 3',5'-cyclic CMP to polypeptidyl UPD-glucose								
	195	1221	C04894	564.8	C00842	305.1	C00941	13.41

#3 from xanthenes to highly glycosylated and aromatic acylated flavonoids								
	167	2108	C16302	642.3	C16290	244.1	C10082	23.76

#4 from xanthenes to C-glycosylated flavonoids								
	159	610.5	C10102	337.7	C10049	222.2	C00799	5.895

#5 from pyrones to biflavonoids								
	157	1120	C10235	502.5	C16191	206.1	C09012	13.34

#M indicates the numbers of molecules in the clusters. Max MW, Ave MW, and Min MW indicate the molecules with the maximum molecular weight, the molecules with the average molecular weight, and the molecules with the minimum molecular weights, respectively, with the respective molecular weights. SD shows the standard deviation of the obtained clusters. Description after the cluster numbers (#1 - #5) represents the group of molecules, in which "from ... to ..." indicates that the molecular structures in the cluster were so diverse that we could not find appropriate words to describe the clusters.

**Figure 5:**
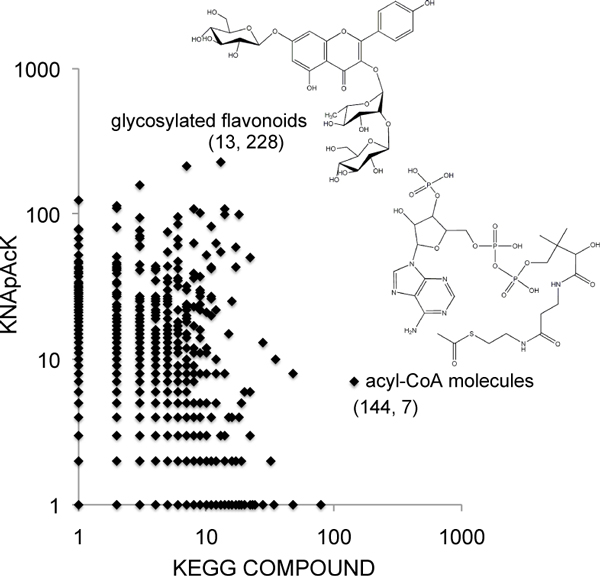
**Scatter plot of the clusters consisting of KEGG COMPOUND and KNApSAcK by KCF-S descriptors**. Each dot represents the QCC clusters obtained by KCF-S descriptors with the weighted Jaccard coefficient >= 0.7 and the clique ratio >= 0.7. The horizontal and vertical axes represent the numbers of KEGG COMPOUND and KNApSAcK molecules in the cluster, respectively.

We further conducted the QCC clustering of the mixed molecules consisting of KEGG COMPOUND and KNApSAcK, with the weighted Jaccard coefficient *>*= 0.7 and the clique ratio *>*= 0.7, and the obtained clusters were plotted onto a scatter diagram (Figure 5). It was clearly shown that KEGG COMPOUND and KNApSAcK contain different distributions of molecular classes. Two example clusters, glycosylated flavonoids and acyl-CoA molecules were presented as such examples in Figure 5. The former cluster consists of 13 and 228 molecules from KEGG COMPOUND and KNApSAcK, and the latter cluster consists of 144 and 7 molecules from KEGG COMPOUND and KNApSAcK, respectively.

### Improved performance in the de novo metabolic pathway reconstruction

As the second application of KCF-S, we tested the proposed descriptors on their abilities to reconstruct metabolic pathways from chemical structures, *i.e*., to predict the enzymatic-reaction likeness of given compound-compound pairs from their chemical fingerprint data, following our previous work [15].

#### Cross-validation experiment to predict enzyme-reaction likeness

We performed the following 5-fold cross-validation. 1) Compound-compound pairs in the gold standard data were split into five subsets of roughly equal sizes. Known reactant pairs were regarded as positive examples, and the other compound-compound pairs as negative examples. 2) Each subset were taken as a test set, and the remaining four subsets as a training set. 3) A predictive model was trained based only on the training set. 4) The prediction scores were computed for compound-compound pairs in the test set. 5) Finally, the prediction accuracy were evaluated over the five folds.

The prediction performance were evaluated by the receiver operating characteristic (ROC) curve, which is a plot of true positives as a function of false positives based on various thresholds, and the precision-recall (PR) curve, which is a plot of precision as a function of recall. The performance were summarized by the area under the ROC curve (AUC) score and the area under the PR curve (AUPR). The parameters involved in the methods were optimized with the AUC score and the AUPR score as the objective functions.

Table 4 shows the resulting AUC scores and AUPR scores for five descriptors: KCF-S, PubChem fingerprint, MACCS fingerprint, and KCF. Among the 4 fingerprints, the proposed KCF-S descriptor achieved the highest AUC and AUPR scores. The higher *k *seems to improve the prediction accuracy to some extent. The KCF-S descriptor outperformed the PubChem fingerprint and the MACCS fingerprint in both AUC and AUPR. This result suggests that the proposed feature vectors are useful.

**Table 4:** Cross validation experiments for predicting the enzymatic-reaction likeness.

Chemical descriptors	Vector dimension	Diff-common L1SVM		Diff-only L1SVM		Baseline		Random	
		**AUC**	**AUPR**	**AUC**	**AUPR**	**AUC**	**AUPR**	**AUC**	**AUPR**

KCF-S k3	53679	0.9841	0.2483	0.9827	0.1872	0.8254	0.0584	0.4981	0.0052
	10000	0.9839	0.2481	0.9824	0.1840	0.8299	0.0594	0.4985	0.0052
	1000	0.9814	0.2269	0.9773	0.1508	0.8397	0.0592	0.5006	0.0053

KCF-S k2	28152	0.9761	0.2144	0.9691	0.1330	0.8122	0.0503	0.4995	0.0053
	10000	0.9763	0.2148	0.9698	0.1366	0.8143	0.0501	0.4997	0.0053
	1000	0.9720	0.2012	0.9596	0.1029	0.8178	0.0481	0.4988	0.0053

KCF-S k1	11133	0.9702	0.1835	0.9620	0.1300	0.8184	0.0776	0.4962	0.0052
	10000	0.9699	0.1835	0.9600	0.1197	0.8187	0.0769	0.4960	0.0052
	1000	0.9676	0.1757	0.9475	0.0868	0.8208	0.0744	0.4963	0.0052

PubChem FP	879	0.9531	0.1341	0.9067	0.0571	0.8883	0.0667	0.5006	0.0052

MACCS FP	164	0.9275	0.0932	0.9097	0.0510	0.8200	0.0336	0.5001	0.0052

ATOM k3	99	0.9532	0.1362	0.9378	0.0703	0.8195	0.0492	0.4983	0.0052

BOND k3	973	0.9773	0.2023	0.9713	0.1319	0.8260	0.0546	0.5001	0.0053

The AUC score of the diff-common feature vector were slightly higher than those of the diff-only feature vector in L1SVM, while the AUPR score of the diff-common feature vector were much higher than those of the diff-only feature vector in L1SVM. This result implies the importance to take into account not only substructure transformation patterns but also common substructures in the reaction prediction. L1SVM outperformed BASELINE, suggesting that supervised learning with the proposed feature vectors is meaningful.

We conducted further analysis to illustrate how much improvement was achieved by KCF-S compared with KCF. Two types of integer vectors were constructed; the one (ATOM descriptor) only contains the ATOM attributes, the other (BOND descriptor) contains the ATOM and BOND attributes. Both attributes can be obtained by using KCF. As the result of the cross-validation experiments, it was clearly shown that the AUC and AUPR scores by KCF-S descriptors were better than those by ATOM and BOND descriptors (Table 4). Obviously, applying KCF-S needs more computational time and memory than KCF. For example, cross-validation experiment needed about 795 seconds and 57 MB memory when using BOND descriptor, whereas about 13,031 seconds and 148 MB memory when using KCF-S descriptor.

#### Examples of newly predicted pathways using KNApSAcK

We applied KCF-S 3k 1000 descriptor to conduct *de novo *metabolic network prediction for all KEGG and KNApSAcK databases. The predicted compound pairs were filtered using the weighted Jaccard coefficient *>*= 0.9, and the connected subnetworks were extracted from the top 10,000 predicted pairs. We manually examined each of the predicted compound pairs to estimate whether or not the one of the pair can be possibly converted to the other in an enzymatic reactions. Taking the 16th largest subnetwork consisting of 181 compounds (mainly flavonoid glycosides) as an example, among the 16290 pairs theoretically obtained, 831 pairs were predicted, and about 100 were considered as positive as the manual examination.

**Figure 6:**
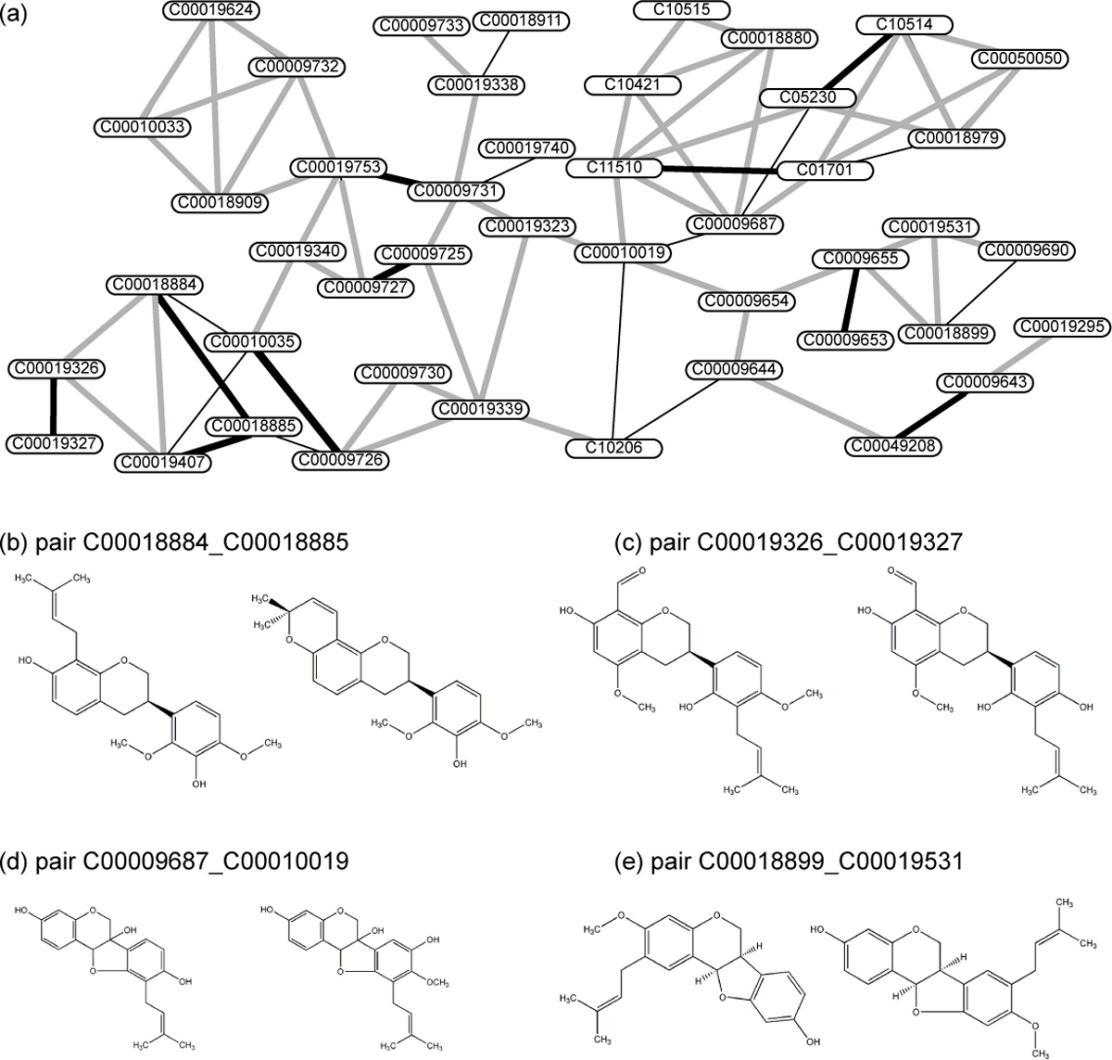
**Example of predicted subnetworks**. Nodes (with ID numbers of KEGG COMPOUND or KNApSAcK) represent molecules. Black bold lines indicate the predicted pairs that were considered as positive after manual examination. Black thin lines and gray lines indicate those that were considered suspicious and negative, respectively. (b) An example pair that was considered as positive, representing a cyclization reaction. (c) Another example pair considered as positive, representing a methylation reaction. (d) An example pair that was considered as suspicious, representing a hydroxylation reaction. (e) An example pair that was considered as negative, representing large rearrangement of carbon skeleton that seems impossible to occur.

Figure 6 shows the 63th largest subnetwork consisting of 45 compounds (mainly prenyl flavonoids), as another example. Among the 990 theoretically defined pairs, 73 pairs were predicted (Figure 6a), of which 10 pairs were considered as positive, 11 pairs were considered as suspicious, and 52 pairs were considered as negative by manual examination. Among the 10 positive pairs, 9 pairs represented cyclization of prenyl flavonoids to form pyrano flavonoids (e.g., Figure 6b), and 1 pair represented methylation (e.g., Figure 6c). Suspicious pairs include hydroxylation on an aromatic ring (e.g., Figure 6d), and negative pairs include isomerations that seems impossible to occur.

## Conclusion

In this study, we introduced a new data structure named KCF-S describing relatively larger biochemical substructures than those defined in KCF format we published in 2003. The main aim of KCF-S is a computationally defined substructures that privides direct links between the names and the substructures in an interpretable way for biochemists. It was shown that the KCF-S helps extract the substructures that are characteristic in any given dataset of molecules. We demonstrated the usefulness of KCF-S for the two applications; structure-based clustering of molecules, and *de novo *metabolic pathway reconstruction. The clusters of molecules obtained by KCF-S were less diverse than those by PubChem and MACCS fingerprints, and were relatively easy to interpret. The improved predictive performance was also achieved by KCF-S for the *de novo *pathway reconstruction. We belive that the KCF-S can also be applied for pharmacogenomic analysis and other studies, taking advantage of the interpretability of the defined substructures.

## Competing interests

The authors declare that they have no competing interests.

## Authors' contributions

MKO conceived of the study, designed the algorithm and drafted the manuscript. YT and YY implemented the algorithms, tested the performance and drafted the manuscript. YM conducted the structure-based clustering of molecules and interpreting the results. TT helped the manual inspection of the output and interpret the results. MKA and SG developed the original KCF format, and helped draft the manuscript. All authors read and approved the final manuscript.

## Supplementary Material

Additional file 1 An additional file contains tables S1-S6 Click here for file

## Declarations

The publication cost for this work was supported by MEXT Kakenhi 25108714.

This article has been published as part of *BMC Systems Biology *Volume 7 Supplement 6, 2013: Selected articles from the 24th International Conference on Genome Informatics (GIW2013). The full contents of the supplement are available online at http://www.biomedcentral.com/bmcsystbiol/supplements/7/S6.
