# Supplementary material for: KCF-S: KEGG Chemical Function and Substructure for improved interpretability and prediction in chemical bioinformatics
Source: BMC Syst Biol. 2013 Dec 13;7(Suppl 6):S2. doi: 10.1186/1752-0509-7-S6-S2 (PMC4029371; doi:10.1186/1752-0509-7-S6-S2)
Supplement: Additional file 1 — An additional file contains tables S1-S6 [file 1752-0509-7-S6-S2-S1.PDF]

Table S1 - Significant substructures in KEGG COMPOUND against KEGG DRUG

The numbers of compounds in the KEGG COMPOUND and KEGG DRUG databases that do or do not contain the respective substructures are counted, and the significant substructures in KEGG COMPOUND against those in KEGG DRUG are listed according to the P-value using Fisher's exact test.

Attributes		KCF-S / annotation	P-value
ATOM	1	R / substituted group	5.7x10^{-135}
	2	O2b / hydroxy phosphate bond	9.4x10^{-134}
	3	O1a / hydroxy	8.9x10^{-122}
	4	O2x / cyclic ether	2.8x10^{-112}
	5	O1c / P-hydroxy	1.0x10^{-89}
BOND	1	C1y-O1a / cyclic secondary alcohol	8.9x10^{-222}
	2	C1y-O2x / cyclic hydroxy ether	1.9x10^{-158}
	3	C1b-O2b / primary alcohol phosphate ester	7.1x10^{-135}
	4	O2-P1 / phosphate ester	2.7x10^{-130}
	5	O2b-P1b / alcohol phosphate ester	6.6x10^{-129}
TRIPLET	1	C1y-C1y-O1a / cyclic secondary alcohol	3.9x10^{-194}
	2	C1y-C1y-O2x / cyclic hydroxy ether	5.5x10^{-189}
	3	C1y-C1y-C1y / cyclic carbon structure	2.9x10^{-180}
	4	C1y-O2x-C1y / cyclic hydroxy ether	1.6x10^{-176}
	5	C1b-C1y-O2x / cyclic hydroxy ether	2.5x10^{-161}
VICINITY	1	C1y(C1y+C1y+O1a) / cyclic secondary alcohol	3.5x10^{-191}
	2	C1y(C1b+C1y+O2x) / cyclic hydroxy ether with branch	2.6x10^{-186}
	3	C1(C1+C1+O1) / secondary alcohol	6.5x10^{-138}
	4	C1(C1+C1+O2) / secondary hydroxy ether	6.1x10^{-124}
	5	P1(O1+O1+O2+O2) / phosphate	1.6x10^{-118}
RING	1	C(C)-C(O)-C(O)-C(O)-C(O)-O / pyranose sugar ring	1.0x10^{-109}
	2	C1(C1)-C1(O1)-C1(O1)-C1(O1)-C1(O2)-O2 / pyranose sugar ring	3.9x10^{-93}
	3	C1y(C1b)-C1y(O1a)-C1y(O1a)-C1y(O1a)-C1y(O2a)-O2x / pyranose sugar ring	6.3x10^{-76}
	4	C(C)-C(O)-C(O)-C(N)-O / furanose sugar ring attached with N	1.3x10^{-51}
	5	C8x-N4y(C1y)-C8y-N5x-C8x-N5x-C8y(N1a)-C8y-N5x / adenine attached with C	2.5x10^{-47}
SKELETON	1	C(O)-C(O)-C(O)-C(O)-C(O)-C(O+O) / hexose sugar	2.5x10^{-84}
	2	C1(O1)-C1(O2)-C1(O1)-C1(O1)-C1(O1)-C1(O2+O2) / hexose sugar	5.4x10^{-77}
	3	C1b(O1a)-C1y(O2x)-C1y(O1a)-C1y(O1a)-C1y(O1a)-C1y(O2a+O2x) / hexopyranose sugar ring O-glycoside	5.1x10^{-73}
	4	C1b(O2b)-C1y(O2x)-C1y(O2b)-C1y(O1a)-C1y(N4y+O2x) / 3,5-substituted pentafuranose sugar ring N-glycoside	9.1x10^{-55}
	5	C1(O2)-C1(O2)-C1(O2)-C1(O1)-C1(N4+O2) pentose sugar N-glycoside / pentafuranose sugar ring N-glycoside	1.0x10^{-53}
INORGANIC	1	O1-P1(O2(C1))(O1)-O1 / alcohol phosphate ester	2.3x10^{-77}
	2	O1-P1(O2(C1))(O1)-O2-P1(O2(C1))(O1)-O1 / di-alkoxy pyrophosphate	1.2x10^{-76}
	3	O-P(O(C))(O)-O-P(O(C))(O)-O / di-alkoxy pyrophosphate	1.2x10^{-76}
	4	O-P(O(C))(O)-O / alcohol phosphate ester	4.5x10^{-75}
	5	O1c-P1b(O2b(C1y))(O1c)-O1c / cyclic secondary alcohol phosphate ester	3.8x10^{-61}

Table S2 - Significant substructures in KEGG DRUG against KEGG COMPOUND

The numbers of compounds in the KEGG DRUG and KEGG COMPOUND databases that do or do not contain the respective substructures are counted, and the significant substructures in KEGG DRUG against those in KEGG COMPOUND are listed according to the P-value using Fisher's exact test.

Attributes		KCF-S / annotation	P-value
ATOM	1	N1y / secondary amine in ring	2.6×10^{-285}
	2	O0 / Undefined oxygen	7.9×10^{-184}
	3	N1c / tertiary amine	1.6×10^{-157}
	4	Z / other atoms	9.2×10^{-151}
	5	S2x / sulfide in ring	9.1×10^{-138}
BOND	1	C1-C8 / alkyl branch on aromatic ring	3.9×10^{-252}
	2	C1x-N1y / cyclic tertiary amine	6.1×10^{-227}
	3	C1b-N1y / cyclic tertiary amine	1.9×10^{-218}
	4	C-X / halide	4.7×10^{-209}
	5	C8-X / aryl halide	1.2×10^{-200}
TRIPLET	1	C8-C8-C8 / aromatic ring	1.7×10^{-308}
	2	C8x-C8x-C8x / aromatic ring	7.2×10^{-295}
	3	C8x-C8y-C8x / aromatic ring	5.4×10^{-248}
	4	C1-C8-C8 / aromatic ring with alkyl branch	3.4×10^{-239}
	5	C1x-C1x-N1y / cyclic tertiary amine	5.1×10^{-216}
VICINITY	1	N1(C1+C1+C1) / tertiary amine	1.2×10^{-280}
	2	C(C+C+N) / tertiary-alkyl amine	5.4×10^{-219}
	3	C8(C8+C8+X) / aryl halide	3.0×10^{-201}
	4	C8(C1+C8+C8) / aromatic ring with alkyl branch	3.2×10^{-201}
	5	N1y(C1b+C1x+C1x) / cyclic tertiary amine	2.0×10^{-192}
RING	1	C-C-C-C-C-C(C) / 6-membered carbon ring with alkyl branch	3.0×10^{-139}
	2	C8-C8-C8-C8-C8-C8(C1) / phenyl ring	5.8×10^{-136}
	3	C-C-N(C)-C-C-N(C) / piperazine ring	1.8×10^{-96}
	4	C-C-C(C)-C-C-C(X) / p-alkyl 6-membered carbon ring halide	8.4×10^{-56}
	5	C8x-C8x-C8x-C8x-C8x-C8y(C1c) / phenyl ring with tertiary carbon	1.6×10^{-49}
SKELETON	1	C(N)-C(N) / ethylene diamine skeleton	1.4×10^{-231}
	2	C1(N1)-C1(N1) / ethylene diamine skeleton	1.6×10^{-181}
	3	C1x(N1y)-C1x(N1y) / cyclic ethylene diamine skeleton	3.7×10^{-116}
	4	C(N)-C(O) / ethanolamine skeleton	1.8×10^{-90}
	5	C(O+O)-C-C-C(O+O) / succinate skeleton	2.1×10^{-72}
INORGANIC	1	O-S(O)(O)-O / sulfate	4.9×10^{-48}
	2	O1d-S4a(O1d)(O1d)-O1d / sulfate	4.9×10^{-48}
	3	O1-S4(O1)(O1)-O1 / sulfate	4.9×10^{-48}
	4	N(C)-S(C)(O)-O / di-alkyl sulfonamide	1.5×10^{-42}
	5	O1d-S4a(C1a)(O1d)-O1d / mesylate	4.4×10^{-38}

Table S3 - Significant substructures in KNApSAcK against KEGG COMPOUND

The numbers of compounds in the KNApSAcK and KEGG COMPOUND databases that do or do not contain the respective substructures are counted, and the significant substructures in KNApSAcK against those in KEGG COMPOUND are listed according to the P-value using Fisher's exact test.

Attributes		KCF-S / annotation	P-value
ATOM	1	O7a / carboxylate ester oxygen	$2.2x10^{-308}$
	2	C7a / carboxylate ester carbon	$3.8x10^{-307}$
	3	O5x / keto oxygen in ring	$1.7x10^{-305}$
	4	O7x / lactone oxygen	$9.4x10^{-292}$
	5	C7x / lactone carbon	$3.1x10^{-270}$
BOND	1	C2-C7 / alkenyl carboxylate ester	$2.6x10^{-318}$
	2	C1-O1 / alcohol	$1.2x10^{-310}$
	3	C7a-O7a / carboxylate ester	$1.6x10^{-308}$
	4	C7a-O6a / carboxylate ester	$3.8x10^{-307}$
	5	C1-C7 / alkyl carboxylate ester	$2.5x10^{-303}$
TRIPLET	1	C2-C2-C7 / alkenyl carboxylate ester	$2.1x10^{-319}$
	2	C2-C7-O6 / alkenyl carboxylate ester	$2.6x10^{-318}$
	3	C2-C7-O7 / alkenyl carboxylate ester	$2.6x10^{-318}$
	4	C8x-C8y-O2a / aryl hydroxy ether	$5.0x10^{-314}$
	5	O6a-C7a-O7a / carboxylate ester	$1.6x10^{-308}$
VICINITY	1	C7(C2+O6+O7) / alkenyl carboxylate ester	$2.6x10^{-318}$
	2	C1(C1+C1+C2) / tertiary carbon attached with alkenyl	$1.3x10^{-316}$
	3	C8y(C8y+C8y+C8y) / condensed aromtic ring	$3.3x10^{-311}$
	4	C8y(C8x+C8y+O2a) / aryl hydroxy ether	$3.8x10^{-306}$
	5	C7(C1+O6+O7) / O-acetyl	$2.5x10^{-303}$
RING	1	C1(C1)-C1(O1)-C1(O1)-C1(O1)-C1(O2)-O2 / pyranose ring	$3.4x10^{-226}$
	2	C(C)-C(O)-C(O)-C(O)-C(O)-O / furanose ring	$5.5x10^{-216}$
	3	C1y(C1b)-C1y(O1a)-C1y(O1a)-C1y(O1a)-C1y(O2a)-O2x / pyranose ring	$1.6x10^{-194}$
	4	C-C-C(C)-C-C(O)-C(O) / 6-membered carbon ring	$1.5x10^{-185}$
	5	C(C)-C(C)-C(O)-C(O)-C(C)-O / 6-membered carbon ring	$1.5x10^{-152}$
SKELETON	1	C1a-C7a(O6a+O7a) / O-acetyl	$8.3x10^{-239}$
	2	C1-C7(O6+O7) / O-acetyl	$8.3x10^{-239}$
	3	C-C(O+O) / O-acetyl	$2.9x10^{-232}$
	4	C(O)-C(O)-C(O)-C(O)-C(O)-C(O+O) / hexopyranose ring	$1.4x10^{-203}$
	5	C1b(O1a)-C1y(O2x)-C1y(O1a)-C1y(O1a)-C1y(O1a)-C1y(O2a+O2x) / hexopyranose ring	$8.5x10^{-154}$
INORGANIC	1	N2(C1+C1+C1)-O3 / tertiary amine N-oxide	$5.1x10^{-13}$
	2	N(C+C+C)-O / tertiary amine N-oxide	$1.6x10^{-11}$
	3	O2(C1)-O2(C1) / dialkyl peroxide	$1.2x10^{-08}$
	4	N2y(C1x+C1x+C1y)-O3a / cyclic tertiary amine N-oxide	$5.1x10^{-08}$
	5	O(C)-O(C) / peroxide	$6.2x10^{-08}$

Table S4 - Significant substructures in KEGG COMPOUND against KNApSAcK

The numbers of compounds in the KEGG COMPOUND and KNApSAcK databases that do or do not contain the respective substructures are counted, and the significant substructures in KEGG COMPOUND against those in KNApSAcK are listed according to the P-value using Fisher's exact test.

Attributes		KCF-S / annotation	P-value
ATOM	1	O2c / pyrophosphate bond	$1.2x10^{-307}$
	2	X / halogen	$8.0x10^{-294}$
	3	Z / other atom	$2.8x10^{-288}$
	4	S2 / sulfide	$3.9x10^{-283}$
	5	C6a / carboxylate carbon	$6.8x10^{-248}$
BOND	1	C8-N1 / aryl amine	$1.7x10^{-318}$
	2	O2c-P1b / pyrophosphate bond	$1.6x10^{-306}$
	3	C8y-N5x / aromatic imine	$4.5x10^{-285}$
	4	C1-N1 / amine	$1.4x10^{-266}$
	5	C8x-N4y / aromatic amine	$1.6x10^{-257}$
TRIPLET	1	P-O-P / pyrophosphate	$4.9x10^{-309}$
	2	P1-O2-P1 / pyrophosphate	$4.9x10^{-309}$
	3	O1c-P1b-O2c / pyrophosphate	$5.9x10^{-306}$
	4	P1b-O2c-P1b / pyrophosphate	$5.9x10^{-306}$
	5	O2b-P1b-O2c / pyrophosphate	$1.3x10^{-305}$
VICINITY	1	P1b(O1c+O1c+O2b+O2c) / pyrophosphate	$4.7x10^{-305}$
	2	N4y(C1y+C8x+C8y) / aromatic tertiary amine	$2.9x10^{-304}$
	3	C1(C1+N4+O2) / aromatic amine aminal	$6.4x10^{-286}$
	4	C(C+C+N) / tertiary carbon amine	$9.6x10^{-280}$
	5	C1y(C1y+C1y+O2b) / tertiary alcohol phosphate	$6.9x10^{-275}$
RING	1	C(C)-C(O)-C(O)-C(N)-O / furanose ring	$4.5x10^{-269}$
	2	C8-N4(C1)-C8(N5)-C8(C8)-N5 / imidazole ring	$2.5x10^{-228}$
	3	C-N(C)-C(N)-C(C)-N / imidazolidine ring	$6.6x10^{-228}$
	4	C8x-N4y(C1y)-C8y(N5x)-C8y(C8y)-N5x / imidazole ring	$4.0x10^{-227}$
	5	C8-N5-C8(N1)-C8(N5)-C8(N4)-N5 / pyrimidine ring	$8.6x10^{-195}$
SKELETON	1	C(O)-C(O)-C(O)-C(O)-C(N+O) / pentose amine	$1.0x10^{-268}$
	2	C8(N1+N5)-C8(N5)-C8(N4+N5) / 2-iminopropanebis(imidamide)	$7.8x10^{-195}$
	3	C8y(N1a+N5x)-C8y(N5x)-C8y(N4y+N5x) / 2-iminopropanebis(imidamide)	$5.1x10^{-189}$
	4	C(N+N)-C(N)-C(N+N) / 2-iminopropanebis(imidamide)	$2.2x10^{-177}$
	5	C1(O2)-C1(O2)-C1(O2)-C1(O1)-C1(N4+O2) / pentose amine	$1.1x10^{-172}$
INORGANIC	1	O1-P1(O2(C1))(O1)-O2-P1(O2(C1))(O1)-O1 / di-alkyl pyrophosphate	$3.4x10^{-246}$
	2	O-P(O(C))(O)-O-P(O(C))(O)-O / di-alkyl pyrophosphate	$3.4x10^{-246}$
	3	O1c-P1b(O2b(C1y))(O1c)-O1c / cyclic secondary alcohol phosphate ester	$4.7x10^{-195}$
	4	O-P(O(C))(O(C))-O / di-alkyl orthophosphate	$5.1x10^{-185}$
	5	O1-P1(O2(C1))(O2(C1))-O1 / di-alkyl orthophosphate	$3.0x10^{-173}$

Table S5 - Significant substructures in KEGG DRUG against KNApSAcK

The numbers of compounds in the KEGG DRUG and KNApSAcK databases that do or do not contain the respective substructures are counted, and the significant substructures in KEGG DRUG against those in KNApSAcK are listed according to the P-value using Fisher's exact test.

Attributes		KCF-S / annotation	P-value
ATOM	1	O3c / S-oxo	$1.0x10^{-309}$
	2	N1y / tertiary amine in ring	$2.5x10^{-294}$
	3	N4 / aromatic amine	$1.1x10^{-283}$
	4	O5a / keto oxygen	$1.1x10^{-282}$
	5	C5a / keto carbon	$1.1x10^{-282}$
BOND	1	C1b-N1b / secondary amine	$3.0x10^{-319}$
	2	C8y-S4a / aryl sulfonate	$5.8x10^{-318}$
	3	C8-S4 / aryl sulfonate	$5.8x10^{-318}$
	4	C5a-N1b / amide	$6.9x10^{-298}$
	5	O3c-S4a / sulfate	$2.6x10^{-292}$
TRIPLET	1	O3-S4-O3 / sulfate	$1.7x10^{-321}$
	2	O3c-S4a-O3c / sulfate	$1.7x10^{-321}$
	3	C8x-C8y-N1b / aryl amine	$2.7x10^{-317}$
	4	C1b-C1b-N1y / cyclic tertiary amine	$2.3x10^{-316}$
	5	C1b-C1b-N1c / non-cyclic tertiary amine	$7.4x10^{-313}$
VICINITY	1	N1(C1+C1+C1) / tertiary amine	$7.2x10^{-320}$
	2	S(C+N+O+O) / sulfonamide	$3.8x10^{-307}$
	3	C8(C8+C8+S4) / aryl sulfonate	$2.4x10^{-291}$
	4	C(N+N+O) / pseudourea	$4.6x10^{-259}$
	5	C8(C8+C8+S2) / aryl sulfide	$1.8x10^{-242}$
RING	1	C-C-N(C)-C-C-N(C) / piperazine ring	$4.3x10^{-253}$
	2	C-C-C(C)-C-C-C(X) / p-alkyl 6-membered carbon ring halide	$2.7x10^{-251}$
	3	C8-C8-C8-C8-C8-C8(C1) / phenyl ring	$5.2x10^{-214}$
	4	C(N)-C(S)-N(C)-C(O) / 2-azetidinone ring	$7.9x10^{-177}$
	5	C-C-C-C-C-C(C) / 6-membered carbon ring	$3.0x10^{-156}$
SKELETON	1	C1x(N1y)-C1x(N1y) / cyclic ethylene diamine	$1.3x10^{-318}$
	2	C(N)-C(O) / ethanolamine	$2.9x10^{-293}$
	3	C1a-C1b(N1c) / N-ethyl	$5.9x10^{-197}$
	4	C(N+S)-C(N)-C(N+O) / diamino-sulfanylpropanamide	$7.9x10^{-177}$
	5	C1(N1+S2)-C1(N1)-C5(N1+O5) / diamino-sulfanylpropanamide	$3.4x10^{-174}$
INORGANIC	1	N(C)-S(C)(O)-O / N,S-dialkyl sulfonamide	$1.3x10^{-178}$
	2	N(C+C)-N(C) / hydrazine	$5.9x10^{-142}$
	3	O-S(C)(O)-O / sulfonate	$1.4x10^{-141}$
	4	N(C)-N(C) / hydrazine	$6.4x10^{-135}$
	5	O-S(O)(O)-O / sulfate	$2.5x10^{-120}$

Table S6 - Significant substructures in KNApSAcK against KEGG DRUG

The numbers of compounds in the KNApSAcK and KEGG DRUG databases that do or do not contain the respective substructures are counted, and the significant substructures in KNApSAcK against those in KEGG DRUG are listed according to the P-value using Fisher's exact test.

Attributes		KCF-S / annotation	P-value
ATOM	1	O7x / lactone oxygen	2.5×10^{-314}
	2	C2a / alkenyl terminus carbon	7.1×10^{-291}
	3	C7x / lactone carbon	4.0×10^{-283}
	4	C2b / alkenyl secondary carbon	3.3×10^{-282}
	5	O7 / lactone oxygen	4.2×10^{-233}
BOND	1	C2-C7 / alkenyl carboxylate ester	9.3×10^{-320}
	2	C2x-C2y / cyclic alkenyl	5.8×10^{-308}
	3	C2b-C2c / non-cyclic alkenyl	9.2×10^{-305}
	4	C7x-O6a / lactone	4.0×10^{-283}
	5	C7x-O7x / lactone	4.0×10^{-283}
TRIPLET	1	C2-C2-C7 / alkenyl caboxylate ester	2.2×10^{-321}
	2	C2-C7-O6 / alkenyl carboxylate ester	9.3×10^{-320}
	3	C2-C7-O7 / alkenyl carboxylate ester	9.3×10^{-320}
	4	C1a-C2c-C2b / non-cyclic alkenyl	1.6×10^{-314}
	5	C-C-C / carbon chain	3.2×10^{-290}
VICINITY	1	C7(C2+O6+O7) / alkenyl carboxylate ester	9.3×10^{-320}
	2	C(C+C+C+C) / quartary carbon	2.3×10^{-308}
	3	C1(C1+C1+C1+C1) / quartary carbon	8.6×10^{-308}
	4	C1y(C1b+C1y+O2x) / cyclic hydroxy ether	2.8×10^{-303}
	5	C8y(C8y+C8y+O2x) / aryl hydroxy ether	1.3×10^{-299}
RING	1	C1y(C1b)-C1y(O1a)-C1y(O1a)-C1y(O1a)-C1y(O2a)-O2x / pyranose ring	2.8×10^{-317}
	2	C-C-C(C)-C-C(O)-C(O) / 6-membered carbon ring	2.2×10^{-149}
	3	C-C(O)-C(C)-C(O)-C(C)-C(O) / 6-membered carbon ring	2.9×10^{-141}
	4	C-C-C(O)-C(C+C)-C(C)-C(C+C) / 6-membered carbon ring	4.8×10^{-141}
	5	C-C(O)-C-C(O)-C(C)-C(O) / 6-membered carbon ring	3.6×10^{-136}
SKELETON	1	C1b(O1a)-C1y(O2x)-C1y(O1a)-C1y(O1a)-C1y(O1a)-C1y(O2a+O2x) / hexopyranose ring	2.7×10^{-273}
	2	C1(O1)-C1(O2)-C1(O1)-C1(O1)-C1(O1)-C1(O2+O2) / hexopyranose ring	1.1×10^{-272}
	3	C1a-C7a(O6a+O7a) / O-acetyl	2.2×10^{-131}
	4	C1-C7(O6+O7) / O-acetyl	2.2×10^{-131}
	5	C-C(O)-C(O)-C(O)-C(O)-C(O+O) / hexopyranose ring	1.7×10^{-130}
INORGANIC	1	O1-O2(C1) / hydroxy peroxide	7.6×10^{-09}
	2	N2b(C2c)-O2a-S4a(O1d)(O1d)-O1d / alkylideneamino oxysulfonic acid	2.2×10^{-08}
	3	O-O(C) / hydroxy peroxide	8.2×10^{-08}
	4	N2(C2)-O2-S4(O1)(O1)-O1 / alkylideneamino oxysulfonic acid	3.8×10^{-07}
	5	N(C)-O-S(O)(O)-O / alkylideneamino oxysulfonic acid	3.8×10^{-07}
